# Preparation of topological modes by Lyapunov control

**DOI:** 10.1038/srep13777

**Published:** 2015-09-08

**Authors:** Z. C. Shi, X. L. Zhao, X. X. Yi

**Affiliations:** 1 School of Physics and Optoelectronic Technology, Dalian University of Technology, Dalian 116024, China; 2 Center for Quantum Sciences and School of Physics, Northeast Normal University, Changchun 130024, China

## Abstract

By Lyapunov control, we present a proposal to drive quasi-particles into a topological mode in quantum systems described by a quadratic Hamiltonian. The merit of this control is the individual manipulations on the boundary sites. We take the Kitaev’s chain as an illustration for Fermi systems and show that an arbitrary excitation mode can be steered into the Majorana zero mode by manipulating the chemical potential of the boundary sites. For Bose systems, taking the noninteracting Su-Schrieffer-Heeger (SSH) model as an example, we illustrate how to drive the system into the edge mode. The sensitivity of the fidelity to perturbations and uncertainties in the control fields and initial modes is also examined. The experimental feasibility of the proposal and the possibility to replace the continuous control field with square wave pulses is finally discussed.

Compared to classical computation, quantum computation has unparallel advantages in solving problems like factoring a large number^1^. However, it is difficult to realize in practice due to decoherence caused by environments. In order to overcome this obstacle, topological quantum computation^2^,^3^,^4^,^5^,^6^ has been proposed, where the ground states are isolated from the rest energy spectrum by gaps, making it robust against perturbations. The topological quantum computation can be performed by braiding non-Abelian anyons^7^,^8^ while the evolution of the system, protected by topology, is described by a nontrivial unitary transformation. The simplest example of the non-Abelian anyons is the Majorana fermions which are self-conjugate quasiparticles and have been extensively studied both theoretically and experimentally. Recently, the Majorana fermions are predicted to exist in fractional quantum Hall system^9^, interface between topological insulator^10^,^11^, topological superconductors^12^,^13^,^14^,^15^,^16^,^17^, solid state system^18^, optical lattices^19^,^20^ and spin chains^21^. Although there are great progress in this field, how to prepare and manipulate Majorana fermions in quantum systems remains challenging.

Generally speaking, a quantum system cannot evolve into a desired state without any quantum controls^22^. While most readers are familiar with the feedback control, here we begin with introducing Lyapunov-based quantum control. The Lyapunov control refers to the use of Lyapunov function to design control fields for manipulating a dynamical system. In quantum mechanics, the evolution of system is governed by the Schrödinger equation and the system state can be described by a time-dependent vector. The Lyapunov function then can be defined as the distance between the time-dependent vector and the target vector. Until now, most studies of Lyapunov control focus on the analysis of largest invariant set^23^,^24^,^25^,^26^, quantum state steering or preparations^27^,^28^. In this work, we extend the application of Lyapunov control and apply it to manipulate many-body system, e.g., driving quasiparticles in a quantum many-body system.

To be specific, by the use of Lyapunov control technique, we present a method to manipulate the topological modes in both Fermi and Bose systems. For a Fermi system described by the Kitaev model, we show how to steer an arbitrary initial mode into the Majorana zero mode by manipulating the chemical potential of the boundary sites. The system can be driven into a special Majorana zero mode localized at one of the boundaries when the initial mode is represented only by creation or annihilation operators. For a Bose system described by the noninteracting Su-Schrieffer-Heeger (SSH) model, the control mechanism is similar to the Fermi system. Nevertheless, due to the vanishing off-diagonal block (pairing terms) in the Hamiltonian, it is impossible to drive an arbitrary superposition of operators with different sites into the target mode except for two special cases, namely, the modes can be solely described by creation (or annihilation) operators or by creation and annihilation operators at same site. An unconventional Lyapunov technique is also explored to achieve the target mode while the conventional Lyapunov control is not effective. The sensitivity of the fidelity to perturbations and uncertainties in the control fields and initial modes is also examined. Finally, we show that the control field can be replaced with square wave pulses, which might make the realization of the control much easier in experiments.

## Results

In this part, we present the main results of this work by showing how well the topological modes can be prepared via the Lyapunov control. The details of calculation and simulation can be found in METHODS. Without loss of generality, we consider a quantum system described by quadratic Hamiltonian,


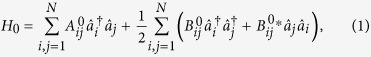


where 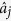 and 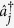 denote the annihilation and creation operators for fermions or bosons at the spatial position *j*. “∗” stands for complex conjugate. The *N* × *N* matrix *A*^0^ (*B*^0^) with elements 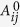 (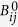) should satisfy 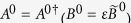 to guarantee the hermicity of *H*_0_, where “∼” denotes transposition, and *ε* = −1 for fermions while *ε* = 1 for bosons. Since the commutation relations of fermions are different from bosons, we will study the control for the Fermi and Boson systems separately.

### Fermi system

We take the 1D Kitaev’s chain of spinless fermions^29^ as an example. The Hamiltonian reads,


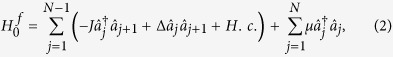


where *J* and Δ are hopping and pairing amplitude, respectively. 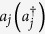 is the fermionic annihilation (creation) operation at site *j*, and *μ* represents the chemical potential. By the pioneering work^29^, one can find that there exist two different topological phases when parameters change. The quantum critical line separating those phases is given by 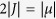 and Δ = 0. To be specific, the parameter satisfying 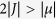 and Δ ≠ 0 is a nontrivial topological phase which can support a Majorana zero mode at the boundaries. In following, we set Δ = 1 and *J* =*μ* = 2 to ensure the existence of the Majorana zero mode in the Kitaev’s chain. The Majorana zero mode can be revealed by solving the secular equation of the BdG Hamiltonian,


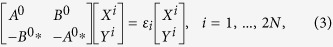


where the elements of matrices *A*^0^ and *B*^0^ are


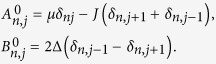


It can be found easily that *X*^*i*^ = *X*^i*^ and *Y*^*i*^ = *Y*^i*^ due to the time-reversal symmetry of the Hamiltonian.

**Figure 1:**
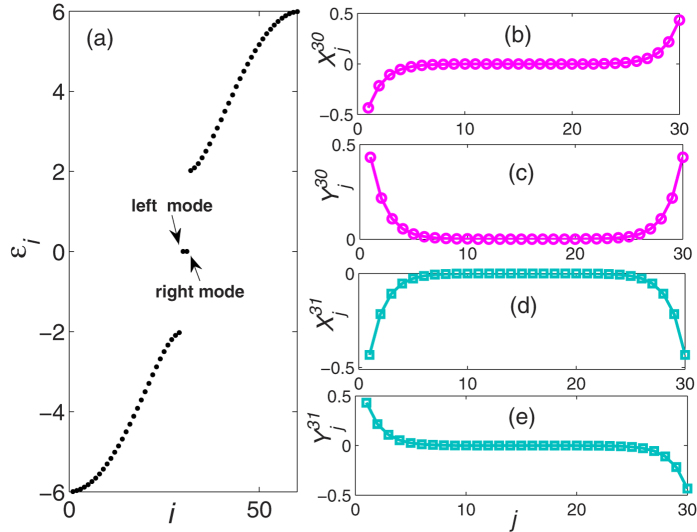
The energy spectrum and spatial distributions of the BdG Hamiltonian 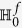 describing the Kitaev’s chain with total number *N* = 30 of sites. We have set the lattice spacing as units. There exists two Majorana modes in the band gap, i.e., the 30*th* and 31*th* eigenmodes. The 30*th* eigenmode is labeled by left mode and the 31*th* is labeled by right mode. (**b**,**c**) are the coefficients *X*^30^ and *Y*^30^ of the left mode, while (**d**,**e**) are the coefficients *X*^31^ and *Y*^31^ of the right mode.

Figure 1(a) demonstrates the eigenvalues of the BdG Hamiltonian, while Fig. 1(b–e) show the distribution of the left and right Majorana zero mode, respectively. As seen in this figure, the Majorana zero mode is located near the two boundary sites of the chain. Taking a chain of length *N* = 30 for concreteness, we show in the following that the Majorana zero mode can be achieved by controlling the chemical potential at the two ends of the Kitaev’s chain. Consider two control Hamiltonians 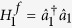 and 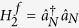, the nonzero elements of matrices *A*^*k*^ given by Eq. (20) corresponding to the control Hamiltonian 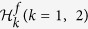 are 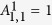 and 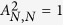.

Suppose that the initial mode is an equally weighted superposition of all sites, namely the initial mode can be expressed as 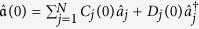 with 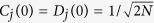. The form of Lyapunov function could be chosen as *V* = *Q*^†^*PQ* and the hermitian matrix *P* could be constructed in the following manner (see methods),


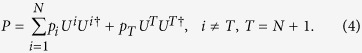


**Figure 2:**
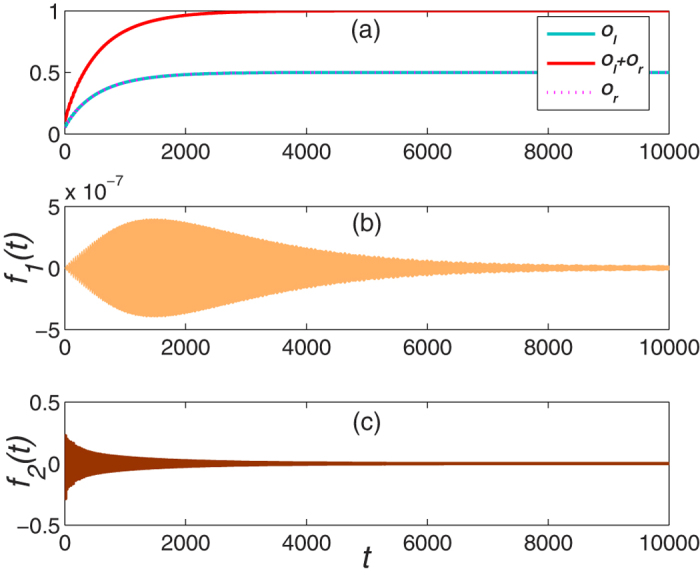
The dynamical evolution of system as a function of time with initial mode 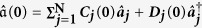. *O*_*l*_ and *O*_*r*_ represent the occupations of the left and right mode, while *O*_*l*_ + *O*_*r*_ approaching unit implies the other quasiparticle modes except the right and left modes are suppressed. (**b**,**c**) denote the dynamical evolution of the control fields *f*_1_(*t*) and *f*_2_(*t*), respectively.

Here *p*_*i*_ = 0, *p*_*T*_ = − 1, and *U*^*T*^ is the target eigenvector. Then the control field becomes 
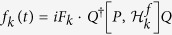
 and we choose *F*_*k*_ = 10 for the numerical calculations. Figure 2 shows the occupations of the left and right mode as a function of evolution time, where the occupation is defined by 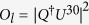 for the left mode, and 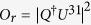 for the right mode. We observe that the initial mode asymptotically converges to the Majorana zero mode with time, and the control fields almost vanish when the system arrives at the target mode. Further simulations show that this proposal works for almost arbitrary initial modes. For example, it can also be driven to the Majorana zero mode when the initial modes are 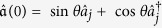 with *θ* ∈ [0, 2*π*].

**Figure 3:**
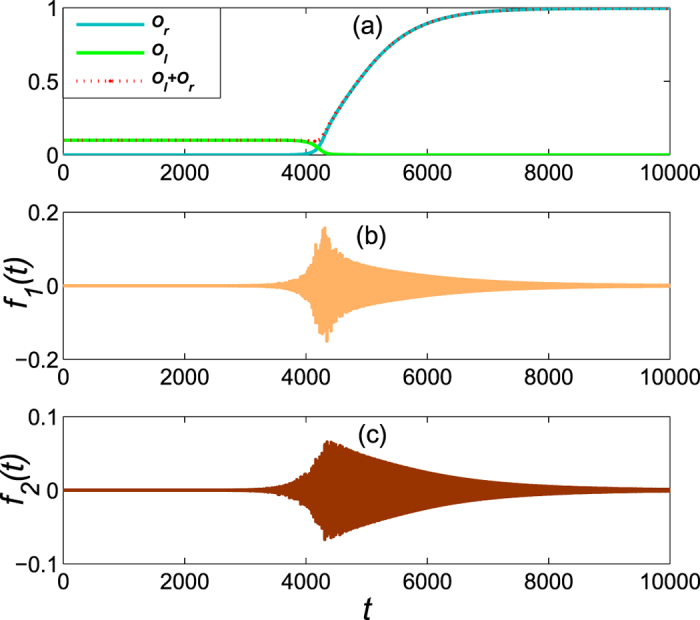
The dynamical evolution of system as a function of time with initial mode 
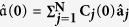
.

For a finite length *N* of the Kitaev’s chain, there exists a weak interaction between the left and right mode with the interaction strength *λ* ∝ *e*^−*N*/*ξ*^ 29, where *ξ* is the coherence length. Obviously, the left and right modes are degenerate when *N*/*ξ*  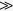  1. Therefore, it is impossible to drive an initial mode into one of the Majorana zero mode individually, if the initial mode includes both the creation and annihilation operators at the same site. However, when the initial mode can be represented by 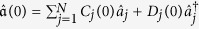 with constraint that *D*_*j*_(0) = 0 if *C*_*j*_(0) ≠ 0 or *C*_*j*_(0) = 0 if *D*_*j*_(0) ≠ 0, it might be possible to drive the initial mode into one of the Majorana zero mode. Figure 3 shows this possibility for driving the system into the right mode while the initial mode is 
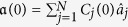
 with 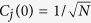. As expected, it converges to the right mode asymptotically.

### Bose system

**Figure 4:**
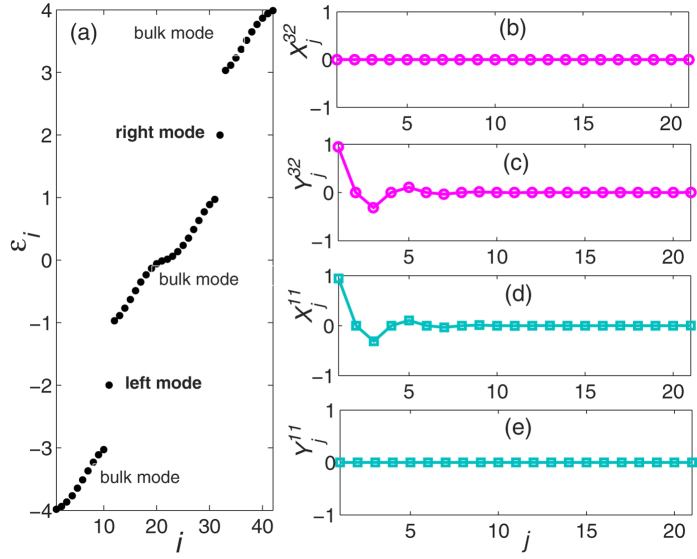
The eigenvalue spectrum and spatial distributions of the Hamiltonian 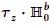 in the SSH model with *N* = 21 sites. Two edge mode are found in the band gap, corresponding to the 11*th* and 32*th* eigenvectors. We label the 11*th* eigenvector as left mode while the 32*th* eigenvector is the right mode. (**b**) and (**c**) are the coefficients *X*^11^ and *Y*^11^ of the left mode while (**d**,**e**) are the coefficients *X*^32^ and *Y*^32^ of the right mode.

For the case of bosons, we take the noninteracting Su-Schrieffer-Heeger (SSH) model^30^ to show the control performance. The Hamiltonian reads


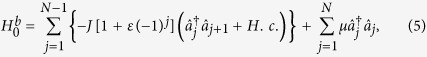


where *ε* is a parameter to change the hoping amplitude *J*, 0 ≤ *ε *≤ 1, and *μ* is the chemical potential. This model can be applied to describe bosons hopping in a double-well 1D optical lattice^31^. The edge mode in the topological band has been shown in Ref. 31, which can be witnessed by the nontrivial Zak phase^32^ of the bulk bands. Thereby it can be taken as the target mode in this control system, and we choose the parameters *J* = 1, *μ* = 2, *N* = 21, and *ε* = 0.3 for the following numerical calculation. Firstly, we present the results of exact diagonalization of 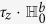 33 (see methods) in Fig. 4(a) and give the coefficients of the edge mode in Fig. 4(b–e). It can be found that the edge mode is located near the first site of the chain, this suggests us to regulate the on-site chemical potential (energy) of site 1 to manipulate the system. Namely, the control Hamiltonian is suggested to be 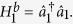 As the Hamiltonian is block diagonal, we could drive the system from an arbitrary initial mode to the target mode for two special cases listed below.

#### Case 1

The initial mode is described by an arbitrary superposition of creation operators or annihilation operators only. Since the annihilation and creation operators that describe quasi-particle modes are decoupled each other, the control system can only converge to the annihilation or creation operators in the target mode, respectively. For the numerical calculations, we choose the initial mode described by a superposition of creation operators 
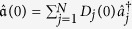
 with 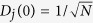. That is, the initial mode contains the creation operators of all sites in this control system. The Lyapunov function is taken as 
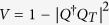
 with 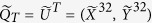, *T* = 32, and the control field is given by 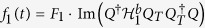 with *F*_1_ =2, where Im(·) denotes the imaginary part of (·).

**Figure 5:**
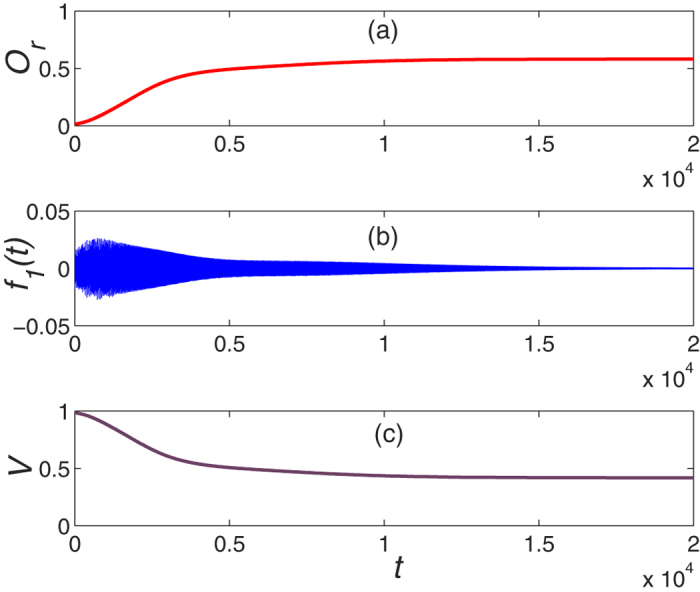
The dynamical evolution of system as a function of time with conventional Lyapunov technique and initial mode 
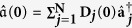
. (**c**) denotes the dynamical behavior of the Lyapunov function *V*.

Figure 5 shows the occupation of right mode as a function of evolution time *t*. It demonstrates that the operator 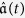 does not completely converge to the right mode since the occupation of the right mode approaches 0.5814. On the other hand, when resolving the characteristic spectrum of the free and control Hamiltonian, one can find that the target mode is controllable for an arbitrary superposition of creation operators. Next, we adopt an implicit Lyapunov-based method to steer an arbitrary initial mode into the right mode^23^, where the Lyapunov function is redefined as


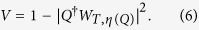


Here, *W*_*T*,*η*(*Q*)_ is a target eigenvector of the matrix 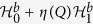 with *η*(*Q*) ∈ 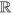 (corresponding to the right mode when *η* = 0, i.e., *W*_*T*, 0_ = *U*^*T*^). The secular equation can be written as,


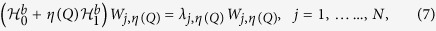


where *λ*_*j*, *η*_ represents the eigenvalues. It returns to the secular equation of the matrix 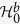 when *η*(*Q*) = 0. The control field can be rewritten as 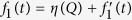, where *η*(*Q*) is implicitly defined as


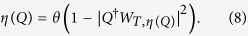


**Figure 6:**
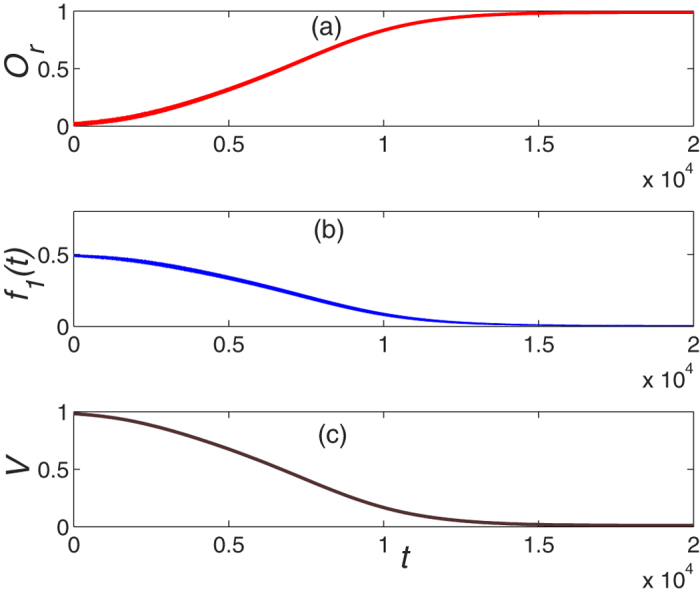
The dynamical evolution of system as a function of time with unconventional Lyapunov technique. The physical parameters are the same to the Fig. 5 except for the Lyapunov function.

Here *θ*(*t*) is a slowly varying real function satisfying *θ*(0) = 0 and *θ*(*t*) > 0 for every *t* > 0. We se*t θ*(*t*) = 0.5 *t* for simplici*t*y. By *t*aking the time derivative of *V*, one can find


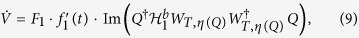


where *F*_1_ is an positive constant. We can choose the control field 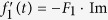 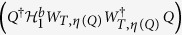 with *F*_1_ = 1 to guarantee 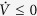. Figure 6 demonstrates the dynamics of occupation of the right mode, we find that it can reach about 0.9887 when completing the control. Hence an arbitrary initial mode can be steered to the right mode by making use of the implicit Lyapunov function.

#### Case 2

The initial mode is an arbitrary superposition of creation and annihilation operators at the same site only, i.e.,


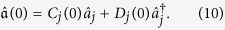


**Figure 7:**
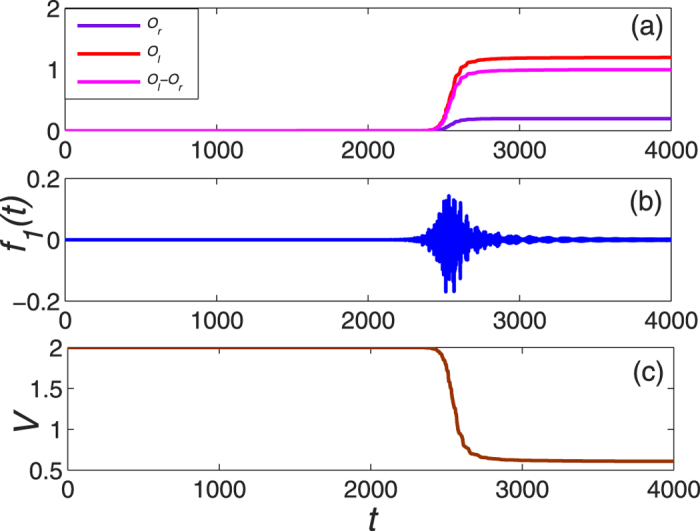
The dynamical evolution of system as a function of time with the Lyapunov function 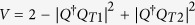
. It can be found that 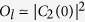 and 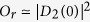 imply the other quasiparticle modes being suppressed.

In this case, the Lyapunov function is chosen a bit different from before, which becomes 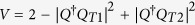 with 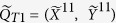 and 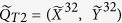. Subsequently, the control field can be straightforwardly taken as 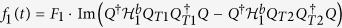. We set 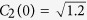, 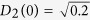 while the other coefficients vanish and *F*_1_ = 1 for numerical calculation. The occupations of the left and right mode are given in Fig. 7. As expected, the Lyapunov function reaches its minimum when the system arrives at the edge mode. The final mode could be approximately written as 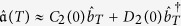, showing that we have realized the edge mode. Note that the occupation difference 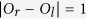 could not guarantee that the final mode converges to the edge mode, which is distinct to the aforementioned cases 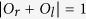. As the evolution of the coefficients of the operator is unitary (see equation (22)) when *B* = 0, the coefficients should satisfy 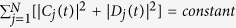, i.e., it is invariant during the evolution. From the numerical calculation, we can find that the final mode can be approximately written as 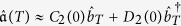, indicating that the coefficients of the other quasiparticle modes almost vanish.

## Discussions

Until now, we have achieved the goal of driving the initial mode of many-body system into a desired quasi-particle mode. The proposal needs to know exactly the system Hamiltonian and the initial mode, as well as to implement precisely the control fields. However, this may be difficult in practice. In experiments, we often encounter uncertainties in the initial modes, perturbations in the control fields, and uncertainties in the Hamiltonian. In previous section, the proposal has been implemented in the Fermi and Bose systems without any perturbations or uncertainties. In following, we discuss the effect of perturbations and uncertainties in the control fields, initial modes and Hamiltonian on the performance of the control.

**Figure 8:**
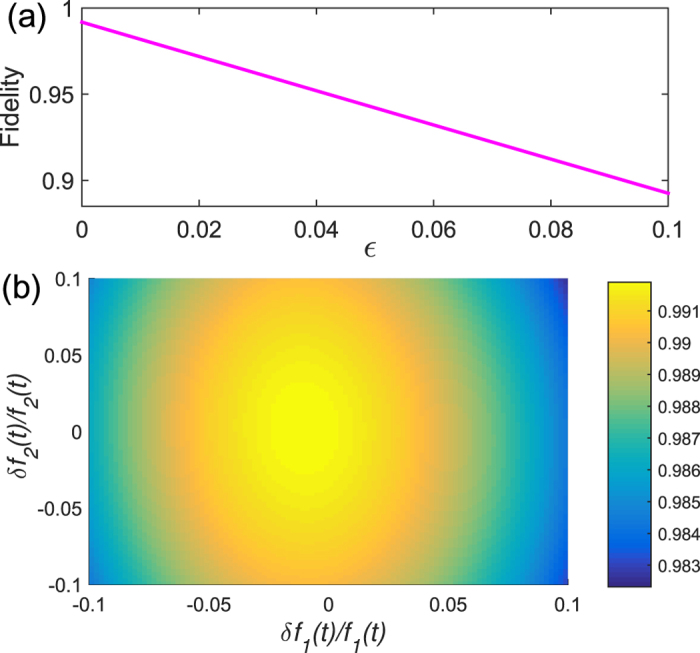
The fidelity versus (**a**) the uncertainties in the initial mode and (**b**) the perturbations in the control fields *f*_1_(*t*) and *f*_2_(*t*). Other parameters are the same as in Fig. 3. The control time is terminated when the fidelity reaches 99.15%. One can observe that the fidelity is still above 98% even though there are 10% perturbations in the control fields.

We first examine the effect of uncertainties in the initial mode and perturbations in the control fields. Taking 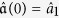 in the Fermi system as the initial mode without uncertainties, we can write the initial mode with uncertainties as 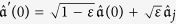 with *ε* quantifying the uncertainties. The dependence of the fidelity on *ε* is plotted in Fig. 8(a). For the control field with perturbations, we write it as 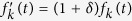 with *f*_*k*_(*t*) representing the perturbationless control field. The dependence of the fidelity on the perturbations is presented in Fig. 8(b). One can find from Fig. 8 that the fidelity is more sensitive to the uncertainties in the initial mode, while it is robust against the perturbations in the control fields. In fact, from the principle of the Lyapunov control, it is suggested that the fidelity of the control process is sensitive to the sign rather than the amplitude of the control fields. This observation can be used to understand the robustness against the perturbations in the control fields.

**Figure 9:**
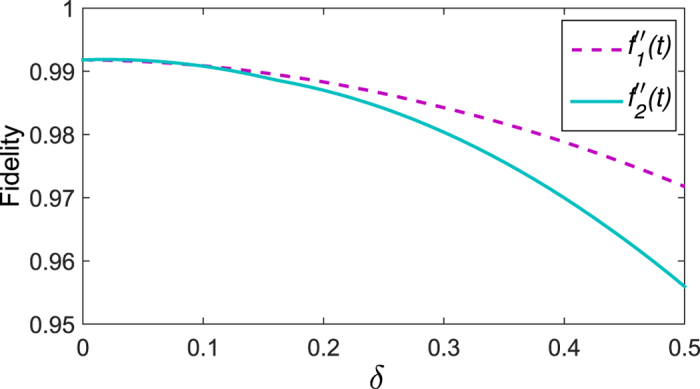
The fidelity versus the chemical potential of nearest-neighbor sites of the boundaries affected by the control fields. We describe this influence by 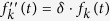. Other parameters are the same as in Fig. 8. *δ* = 0.5 means that the value of control fields on the nearest-neighbor site is the half of control fields on the boundary sites.

In a more realistic circumstance, individual controls on the boundary sites are difficult to implement, which means that the control on the boundary sites might affect the on-site chemical potential of their nearest neighbors. Suppose that the chemical potential of the nearest-neighbor sites, which is affected by the control fields, can be characterized by 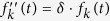, i.e., the on-site chemical potential of 2*nd* and (*N* − 1)*th* site are replaced by 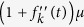. The results in Fig. 9 suggest that the fidelity keeps high even though the control fields have influences on the nearest-neighbor sites.

**Figure 10:**
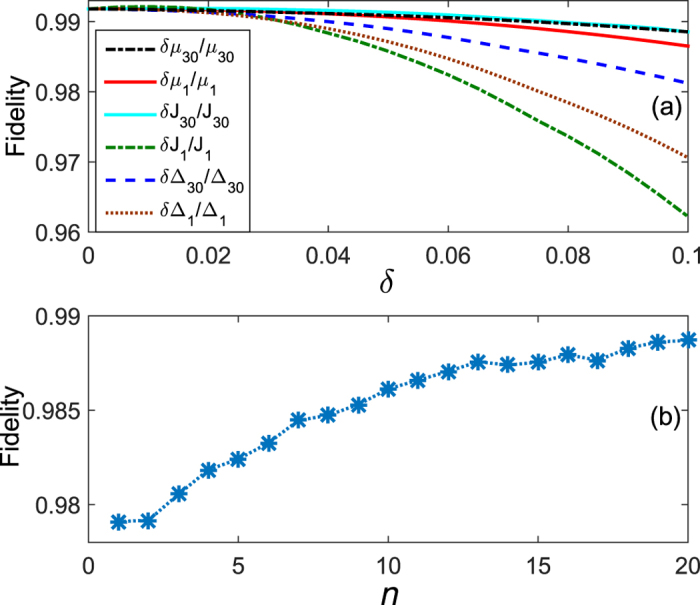
The effect of uncertainties in the Hamiltonian on the fidelity. The influence of boundary Hamiltonian is depicted in (**a**). Each point is an average over 30 simulations in (**b**). The horizontal axis denotes the number of perturbations at each instance of time in the Kitaev’s chain. Other parameters are the same as in Fig. 8.

On the other hand, the Lyapunov control requires to know the system Hamiltonian exactly, which may be difficult in practice. One then may ask how does the control performance change if there exist uncertainties in the Hamiltonian. We now turn to study this problem. The Hamiltonian with uncertainties can be written as 
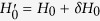
. Here, *δH*_0_ denotes the deviation (called uncertainties) of the Hamiltonian in the control system. This deviation might manifest in the hopping amplitude *J*, pairing Δ, or the chemical potential *μ*. As the control is exerted on the boundary sites only, we study the deviation in the boundary sites and the bulk sites, separately. Figure 10(a) shows the fidelity as a function of the deviations in the boundary Hamiltonian, 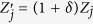 (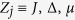, where *j* = 1, *N*). It finds that the deviations caused by the boundary Hamiltonian do not have a serious impact on the fidelity. When the deviation happens in the bulk sites, for example, the on-site chemical potential 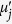 of the bulk sites is replaced with 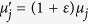 (note that site *j* is randomly chosen from the bulk, and *ε* is an random number, *ε* *∈* [ − 0.02, 0.02]), we consider *n* (*n* = 1, …, 20) uncertainties appearing simultaneously at each instance of evolution time. In other words, we simulate *n* fluctuations for the on-site chemical potentials, where each fluctuation is generated for a randomly chosen site *n*, the value of fluctuations for chosen sites is randomly created and denoted by *ε*. By performing the extensive numerical simulations, we demonstrate the results in Fig. 10(b). It can be found that the quantum system is robust against small uncertainties since the fidelity is always larger than 97.9%. An interesting observation is that with the number of fluctuations increasing, the fidelity increases. This can be understood as follows. Firstly, the small deviations cannot close the gaps in the topological system, thus the fidelity would not deteriorate sharply. Secondly, although more uncertainties participate in the control procedure, the average of the uncertainties almost approaches zero as the average of the random number *ε* is zero.

Since the form of control field generally takes 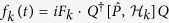, the amplitude of the control fields may change fast with time, which increases the difficulty in the realizations. It is believed that the square wave pulses can be readily achieved in experiments. Therefore we try to take the square wave pulses instead of 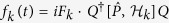 for the control field. The principle to design the square wave pulses should satisfy,


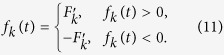


**Figure 11:**
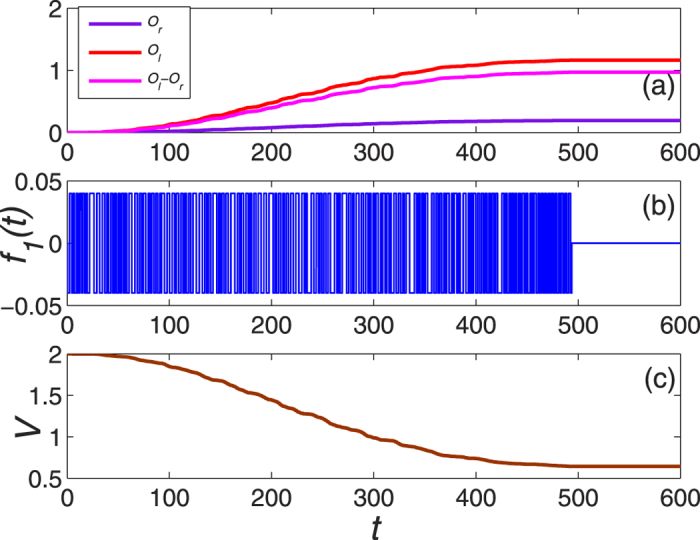
The dynamical evolution of system as a function of time with the square wave pulses.

As an example, we focus on the Bose system whose parameters are the same as in Fig. 7 except that the control field *f*_1_(*t*) is replaced by the equation (11) with 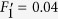. Figure 11 demonstrates the results for the square wave pulses of the control field and it can also achieve the edge mode eventually. On the other hand, we find that convergence time is shortened as well. Of course, the square wave pulses of the control fields can also be applied to the Fermi system.

Finally, we would like to discuss on the experimental feasibility for the present control protocols. The SSH model can be experimentally realized by ^87^Rubidium atoms^34^ in 1D double-well optical lattice^35^. The implementations of Lyapunov control require to perform operations defined by the control Hamiltonians with strengths defined by the control fields. In our case, the control Hamiltonians are the particle number operators of the boundary sites, and the control can be experimentally realized by manipulating the on-site chemical potentials of the boundary sites. The realization of Kitaev’s chain requires spinless fermions, which can be prepared in an optical lattice by trapping the fermions and the BEC reservoir with Feshbach molecules (the couplings between them can be induced by an rf-pulse)^36^. By driving the fermions with Raman laser to produce a strong effective coupling, the system in this situation is equivalent to the Kitaev’s chain. In order to realize the control Hamiltonians, one can adopt additional lasers to control the chemical potentials of the boundary sites, where the intensity of lasers is simulated by square wave pulses (e.g., see *f*_1_(*t*) in Fig. 11(b)). In addition, we can realize the effective Kitaev’s chain in the quantum-dot-superconductor system^37^, a linear array with quantum dots linked by s-wave superconductors with normal and anomalous hoppings. In this system, the chemical potential in each quantum dot can be controlled individually by gate voltages with a high degree of precision. Alternatively, the Kitaev’s chain can also be achieved in the system which consists of a strong spin-orbit interaction semiconductor nanowire (in the low density limit) coupling to a superconductor in magnetic field^38^,^39^. Then the boundary chemical potential can be controlled by local gates^40^,^41^. Most recently, the observation of Majorana fermions in this system has also been observed in experiments^41^,^42^.

In summary, we present a scheme to prepare quasi-particle mode by Lyapunov control in the both Fermi and Bose systems. For the Fermi system, we choose the Kitaev’s model as an illustration and specify the Majorana zero mode as the target mode. The results show that by controlling the chemical potential at the two boundary sites, the system can be driven asymptotically into one of the Majorana zero mode such as the right mode. In contrary, the situation for bosons is different due to the commutation relations. As an example, in the noninteracting SSH model, we show how to prepare the edge mode by the control fields. In particular, we apply the implicit Lyapunov-based technique to the boson system which provides us with a new way to steer the bosons. The robustness of the fidelity against perturbations and uncertainties is also examined. Finally, we try to replace the control fields with square wave pulses, which might help realize the control fields more easily in experiments since it is difficult to apply a fast time-varying control fields in practice.

## Methods

In this part, we give the derivation of the control scheme, starting with the quadratic Hamiltonian,


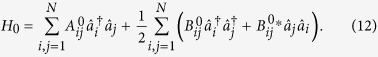


For the case of fermions, we denote the Hamiltonian by 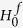, i.e., 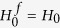. The operators obey the anticommutation relations: 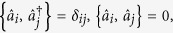 and 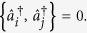 Define a time-dependent fermionic operator,


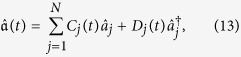


where the operators 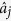 and 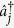 are time-independent while the coefficients are time-dependent. It is easy to check that 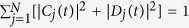 according to the anti-commutation relation 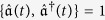. In the Heisenberg picture, the evolution of this operator satisfies (*ħ* = 1),


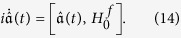


After a brief algebraic operation, the equation becomes


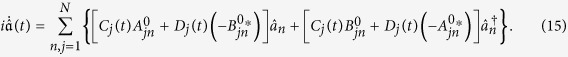


The evolution of coefficients *C*_*j*_(*t*) and *D*_*j*_(*t*) then can be written in a compact form of matrix,


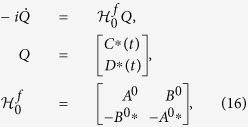


where 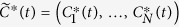 and 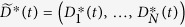. We use the Gothic letter 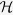
_0_ to denote the matrix in equation (16) corresponding to the Hamiltonian *H*_0_ in equation (12) for simplicity hereafter.

For the Fermi system, the quadratic Hamiltonian can be rewritten as 
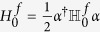
 up to a constant factor (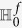 called Bogoliubov-de-Gennes(BdG) Hamiltonian), where 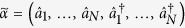. Clearly, 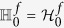. In fact, the equation (16) is actually the BdG-Schrödinger equation^43^, where *Q* is the quasi-particle wave function in the Nambu representation. One can claim that if *ε*_*l*_ is an eigenvalue of 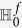 with corresponding eigenvector 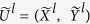, *l* = 1, …, *N*:


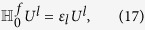



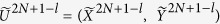
 is also an eigenvector with eigenvalue −*ε*_*l*_, i.e.,


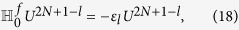


where *X*^l*^ = *Y*^2*N* + 1 − *l*^, *Y*^l*^ = *Y*^2*N* + 1 − *l*^, 
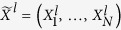
, and 
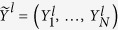
. Thus the eigenvalues come in pairs ±*ε*_*l*_ for the BdG Hamiltonian 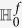
33. Diagonalizing the BdG Hamiltonian, the quasi-particles can be represented by annihilation (creation) operators 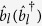,


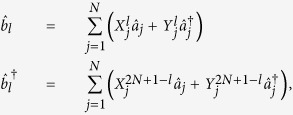


where *l* = 1, …, *N*. In terms of the quasi-particle modes, the Hamiltonian can be written as 
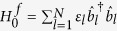
, where 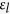 are the energy of the quasi-particle 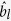.

Let one of the quasi-particle modes be the target mode which we want to prepare, e.g., 
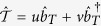
 where *u* and *v* are arbitrary constants. 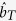 and 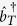 are the annihilation and creation operators of the target mode, respectively. The goal is to design control fields that can drive any initial modes to the target one. It should be noticed that we cannot choose the target arbitrarily because it depends on the free Hamiltonian. In other words, we need a stationary target mode which does not evolve under the free Hamiltonian. As the edge mode is robust against perturbations, we focus on the preparation of it. The evolution described by the equation (16) is unitary since 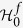 is hermitian. As a result the sum 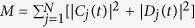 remains unchanged during the time evolution. To make the calculation clear, we write the target mode as 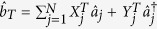, in which 
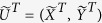
 is an eigenvector of the BdG Hamiltonian 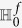, meanwhile it is also a solution of equation (16). Namely, 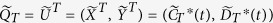, where 
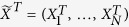
 and 
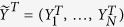
. Assume that there are *K* control Hamiltonians 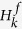 for the system in quadratic form: 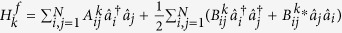, *k* = 1, …, *K*. Together with the original Hamiltonian, the equation of motion for the coefficients in the operator 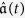 becomes


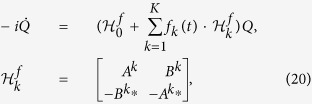


where *f*_*k*_(*t*) is the control field.

There are many choices for the Lyapunov functions, for example, 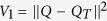, 
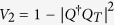
, and *V*_3_ = *Q*^†^*PQ*. Here, 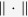 denotes the norm. Those Lyapunov functions are nonnegative and reach the minimum when the system arrives at the target. Apparently, different Lyapunov functions lead to different invariant set and different characteristics of convergence. In following, we choose *V* = *Q*^†^*PQ* as the Lyapunov function to show how our scheme works while the analysis for other Lyapunov functions are similar to it. To this end, it is instructive to deduce the first-order time derivative of the Lyapunov function,


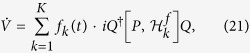


where we have set 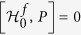 by properly constructing the matrix *P*. In order to make the time derivative of *V* non-positive, one can design the control fields in the following style: 
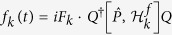
 with *F*_*k*_ > 0. Strictly speaking, the quantum system converges to the invariant set determined by the La Salle’s invariance principle, equivalent to the solution 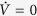.

Note that the commutation relations for bosons: 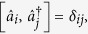 and 
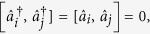
 are different from fermions. Keeping this difference in mind and by an analysis similar to the case of fermions, one can obtain a dynamical evolution of operator 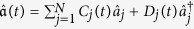 with 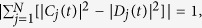



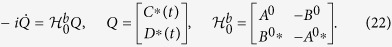


In this case, the matrix of BdG Hamiltonian 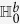 is 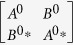. Therefore, we can find that 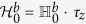, where 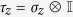, *σ*_*z*_ is Pauli matrix and II is the *N* × *N* identity matrix. The dynamics of coefficients are not unitary in general except for *B*^0^ = 0. For this special situation, the control mechanism is analogous to the case of fermions.

## Additional Information

**How to cite this article**: Shi, Z. C. *et al.* Preparation of topological modes by Lyapunov control. *Sci. Rep.* **5**, 13777; doi: 10.1038/srep13777 (2015).

## References

[b1] Shor P. Algorithms for quantum computation: Discrete logarithms and factoring. IEEE Press, Los Alamitos, 124–134 (1994). 10.1109/SFCS.1994.365700.

[b2] Bonderson P., Freedman M. & Nayak C. Measurement-only topological quantum computation. Phys. Rev. Lett. 101, 010501 (2008). 18764095 10.1103/PhysRevLett.101.010501

[b3] Nayak C. *et al.* Non-Abelian anyons and topological quantum computation. Rev. Mod. Phys. 80, 1083 (2008).

[b4] Bonderson P. Measurement-only topological quantum computation via tunable interactions. Phys. Rev. B 87, 035113 (2013). 10.1103/PhysRevLett.101.010501 18764095

[b5] Akhmerov A. R. Topological quantum computation away from the ground state using Majorana fermions Phys. Rev. B 82, 020509 (2010).

[b6] Mong R. S. K. *et al.* Universal topological quantum computation from a superconductor-abelian quantum hall heterostructure. Phys. Rev. X 4, 011036 (2014).

[b7] Kitaev A. Y. Fault-tolerant quantum computation by anyons. Ann. Phys. 303, 2–30 (2003).

[b8] Kitaev A. Y. Anyons in an exactly solved model and beyond. Ann. Phys. 321, 2–111 (2006).

[b9] Read N. & Green D. Paired states of fermions in two dimensions with breaking of parity and time-reversal symmetries and the fractional quantum Hall effect. Phys. Rev. B 61, 10267 (2000).

[b10] Hasan M. Z. & Kane C. L. Colloquium: Topological insulators. Rev. Mod. Phys. 82, 3045 (2010).

[b11] Qi X. L. & Zhang S. C. Topological insulators and superconductors. Rev. Mod. Phys. 83, 1057 (2011).

[b12] Fu L. & Kane C. L. Superconducting proximity effect and majorana fermions at the surface of a topological insulator. Phys. Rev. Lett. 100, 096407 (2008). 18352737 10.1103/PhysRevLett.100.096407

[b13] Sau J. D., Lutchyn R. M., Tewari S. & Sarma S. D. Generic new platform for topological quantum computation using semiconductor heterostructures. Phys. Rev. Lett. 104, 040502 (2010). 20366693 10.1103/PhysRevLett.104.040502

[b14] Cheng M., Lutchyn R. M., Galitski V. & Sarma S. D. Tunneling of anyonic Majorana excitations in topological superconductors. Phys. Rev. B 82, 094504 (2010).

[b15] Seradjeh B. & Grosfeld E. Unpaired Majorana fermions in a layered topological superconductor. Phys. Rev. B 83, 174521(2011).

[b16] Stoudenmire E. M., Alicea J., Starykh O. A. & Fisher M. P. A. Interaction effects in topological superconducting wires supporting Majorana fermions. Phys. Rev. B 84, 014503 (2011).

[b17] Biswas R. R. Majorana fermions in vortex lattices. Phys. Rev. Lett. 111, 136401 (2013). 24116796 10.1103/PhysRevLett.111.136401

[b18] Alicea J. New directions in the pursuit of Majorana fermions in solid state systems. Rep. Prog. Phys, 75, 076501 (2012). 22790778 10.1088/0034-4885/75/7/076501

[b19] Kraus C. V., Diehl S., Zoller P. & Baranov M. A. Preparing and probing atomic Majorana fermions and topological order in optical lattices. New J. Phys. 14, 113036 (2012).

[b20] Mei F. *et al.* Creation, manipulation and detection of Majorana fermions with cold atoms in optical lattice. *arXiv*:1204.3974 (2012).

[b21] Niu Y. *et al.* Majorana zero modes in a quantum Ising chain with longer-ranged interactions. Phys. Rev. B 85, 035110 (2012).

[b22] D’Alessandro D. Introduction to Quantum Control and Dynamics (Taylor and Francis Group, Boca Raton, 2007).

[b23] Beauchard K., Coron J. M., Mirrahimi M. & Rouchon P. Implicit Lyapunov control of finite dimensional Schrödinger equations. Systems and Control Letters. 56, 388–395 (2007).

[b24] Kuang S. & Cong S. Lyapunov control methods of closed quantum systems. Automatica 44, 98–108 (2008).

[b25] Coron J. M., Grigoriu A., Lefter C. & Turinici G. Quantum control design by Lyapunov trajectory tracking for dipole and polarizability coupling. New J. Phys. 11, 105034 (2009).

[b26] Wang X. T. & Schirmer S. G. Analysis of Lyapunov method for control of quantum states. IEEE Transactions on Automatic Control 55, 2259–2270 (2010).

[b27] Yi X. X., Huang X. L., Wu C. F. & Oh C. H. Driving quantum systems into decoherence-free subspaces by Lyapunov control. Phys. Rev. A 80, 052316 (2009).

[b28] Wang X. T. & Schirmer S. G. Entanglement generation between distant atoms by Lyapunov control. Phys. Rev. A 80, 042305 (2009).

[b29] Kitaev A. Y. Unpaired Majorana fermions in quantum wires. Phys. Usp. 44, 131 (2001).

[b30] Heeger A. J., Kivelson S., Schrieffer J. R. & Su W. P. Solitons in conducting polymers. Rev. Mod. Phys. 60, 781 (1988).

[b31] Strabley J. S., Anderlini M., Jessen P. S. & Porto J. V. Lattice of double wells for manipulating pairs of cold atoms. Phys. Rev. A 73, 033605 (2006).

[b32] Zak J. Berry’s phase for energy bands in solids. Phys. Rev. Lett. 62, 2747 (1989). 10040078 10.1103/PhysRevLett.62.2747

[b33] Blaizot J. P. & Ripka G. Quantum Theory of Finite Systems (MIT Press, Cambridge, MA, 1986).

[b34] Bücker R. *et al.* Twin-atom beams. Nature Phys. 7, 608–611 (2011).

[b35] Barnett R. Edge-state instabilities of bosons in a topological band. Phys. Rev. A 88, 063631 (2013).

[b36] Jiang L. *et al.* Majorana fermions in equilibrium and in driven cold-atom quantum wires. Phys. Rev. Lett. 106, 220402 (2011). 21702583 10.1103/PhysRevLett.106.220402

[b37] Sau J. D. & Sarma S. D. Realizing a robust practical Majorana chain in a quantum-dot-superconductor linear array. Nat. Comm. 3, 964 (2012). 10.1038/ncomms1966 22805571

[b38] Lutchyn R. M., Sau J. D. & Sarma S. D. Majorana fermions and a topological phase transition in semiconductor-superconductor heterostructures. Phys. Rev. Lett. 105, 077001 (2010). 20868069 10.1103/PhysRevLett.105.077001

[b39] Oreg Y., Refael G. & Oppen F. V. Helical liquids and majorana bound states in quantum wires. Phys. Rev. Lett. 105, 177002 (2010). 21231073 10.1103/PhysRevLett.105.177002

[b40] Alicea J. *et al.* Non-Abelian statistics and topological quantum information processing in 1D wire networks. Nature Phys. 7, 412–417 (2011).

[b41] Das A. *et al.* Zero-bias peaks and splitting in an AlCInAs nanowire topological superconductor as a signature of Majorana fermions. Nature Phys. 8, 887–895 (2012).

[b42] Mourik V. *et al.* Signatures of Majorana Fermions in Hybrid Superconductor-Semiconductor Nanowire Devices. Science, 336, 1003–1007 (2012). 22499805 10.1126/science.1222360

[b43] de Gennes P. G. Superconductivity of Metals and Alloys (W. A. Benjamin, New York, 1966).

